# Genetic causal relationship between immune diseases and migraine: a Mendelian randomization study

**DOI:** 10.3389/fimmu.2024.1376698

**Published:** 2024-04-08

**Authors:** Guanglu Li, Shaojie Duan, International Headache Genetics Consortium (IHGC), Verneri Anttila, Tao Zheng, Tiantian Zhu, Baoquan Qu, Lei Liu, Zunjing Liu

**Affiliations:** ^1^ Graduate School of Beijing University of Chinese Medicine, Beijing, China; ^2^ Department of Neurology, China-Japan Friendship Hospital, Beijing, China; ^3^ Department of Geriatrics, Taizhou Central Hospital (Taizhou University Hospital), Taizhou, China; ^4^ Beijing Tiantan Hospital, Capital Medical University, Beijing, China; ^5^ Department of Neurology, Peking University People’s Hospital, Beijing, China

**Keywords:** migraine, immune diseases, causal association, Mendelian randomization, genetic correlation

## Abstract

**Background:**

Migraine has an increased prevalence in several immune disorders, but genetic cause-effect relationships remain unclear. Mendelian randomization (MR) was used in this study to explore whether immune diseases are causally associated with migraine and its subtypes.

**Methods:**

We conducted a two-sample bidirectional multivariate Mendelian randomization study. Single-nucleotide polymorphisms (SNP) for six immune diseases, including rheumatoid arthritis (RA), systemic lupus erythematosus (SLE), type 1 diabetes mellitus (T1D), allergic rhinitis (AR), asthma and psoriasis, were used as genetic instrumental variables. Summary statistics for migraine were obtained from 3 databases: the International Headache Genetics Consortium (IHGC), UK Biobank, and FinnGen study. MR analyses were performed per outcome database for each exposure and subsequently meta-analyzed. Reverse MR analysis was performed to determine whether migraine were risk factors for immune diseases. In addition, we conducted a genetic correlation to identify shared genetic variants for these two associations.

**Results:**

No significant causal relationship was found between immune diseases and migraine and its subtypes. These results were robust with a series of sensitivity analyses. Using the linkage disequilibrium score regression method (LDSC), we detected no genetic correlation between migraine and immune diseases.

**Conclusion:**

The evidence from our study does not support a causal relationship between immune diseases and migraine. The mechanisms underlying the frequent comorbidity of migraine and several immune diseases need to be further elucidated.

## Introduction

Migraine is a common disabling neurological disorder, affecting more than 14.4% of the global population (1, 2). Migraine is the leading cause of years lived with disability (YLD) and disability-adjusted life years (DALYs) (3), particularly in young women (4, 5).

Migraine is now recognized as a complex brain disorder closely related to genetics, with the heritability being 42% (6). Significant progress has been achieved in the study of migraine genetics. Key proteins identified in monogenic migraine significantly increase susceptibility to cortical spreading depression (CSD), which is considered critical for triggering migraine aura (7). Multiple migraine Genome-wide association studies (GWAS) have identified over 180 risk loci in the human genome, confirming that migraine is a predominantly polygenic neurovascular disorder (8–11). In addition, examining the shared genetic background of migraine and its comorbidities and traits may provide insights into the genetics of migraine. Several genetic correlation and Mendelian randomization (MR) studies based on GWAS data have demonstrated the genetic susceptibility between migraine and various disorders, including psychiatric disorders (12), ischaemic stroke (13), and coronary artery disease (14).

Associations between migraine and multiple immunological diseases have been shown in epidemiological studies. Many immune diseases are more common in young females (15, 16), which is very similar to migraine - the prevalence of migraine in adult females is 3-4 times higher than in males but only rarely after 50 years of age (1, 17, 18), suggesting that migraine and immune disorders may share a common genetic etiologic basis. Observational study results are vulnerable to confounding factors, including demographics, environmental exposures, and self-reported diagnostic uncertainty, therefore results should be carefully interpreted. The existence and direction of a causal relationship between migraine and immunological diseases are still controversial. Investigating the relationship between immunological diseases and migraine may provide insights into immune disorders in migraine attacks.

MR offers a means of utilizing genetic variants (i.e., single nucleotide polymorphisms, SNPs) for instrumental variables (IVs) to estimate the potential causality of modifiable risk factors for specific diseases (19, 20). Since genetic variants follow the principle of random assignment of alleles to offspring at meiosis, this process is similar to the randomization process in Randomized Controlled Trials. Genetic variants preceded the onset of exposure and outcome, thus excluding reverse causality (21, 22).

Large-scale publicly available GWAS data provide substantial and reliable genetic variation for MR studies. Recently, MR methods have been widely used to illuminate causal relationships between commonly associated traits/diseases, which opens new pathways for hypothesis-driven mechanistic studies and well-rationalized clinical trial design.

Based on population GWAS summary-level data obtained from large consortiums, we first estimated the genetic correlations between migraine and six immune disorders, including rheumatoid arthritis (RA), systemic lupus erythematosus (SLE), type 1 diabetes mellitus (T1D), allergic rhinitis (AR), asthma, and psoriasis. Then, we used an MR analysis to investigate the causality between them. For each exposure, MR analyses were performed per migraine outcome database and were subsequently meta-analyzed.

## Materials and methods

### Study design

**Figure 1:**
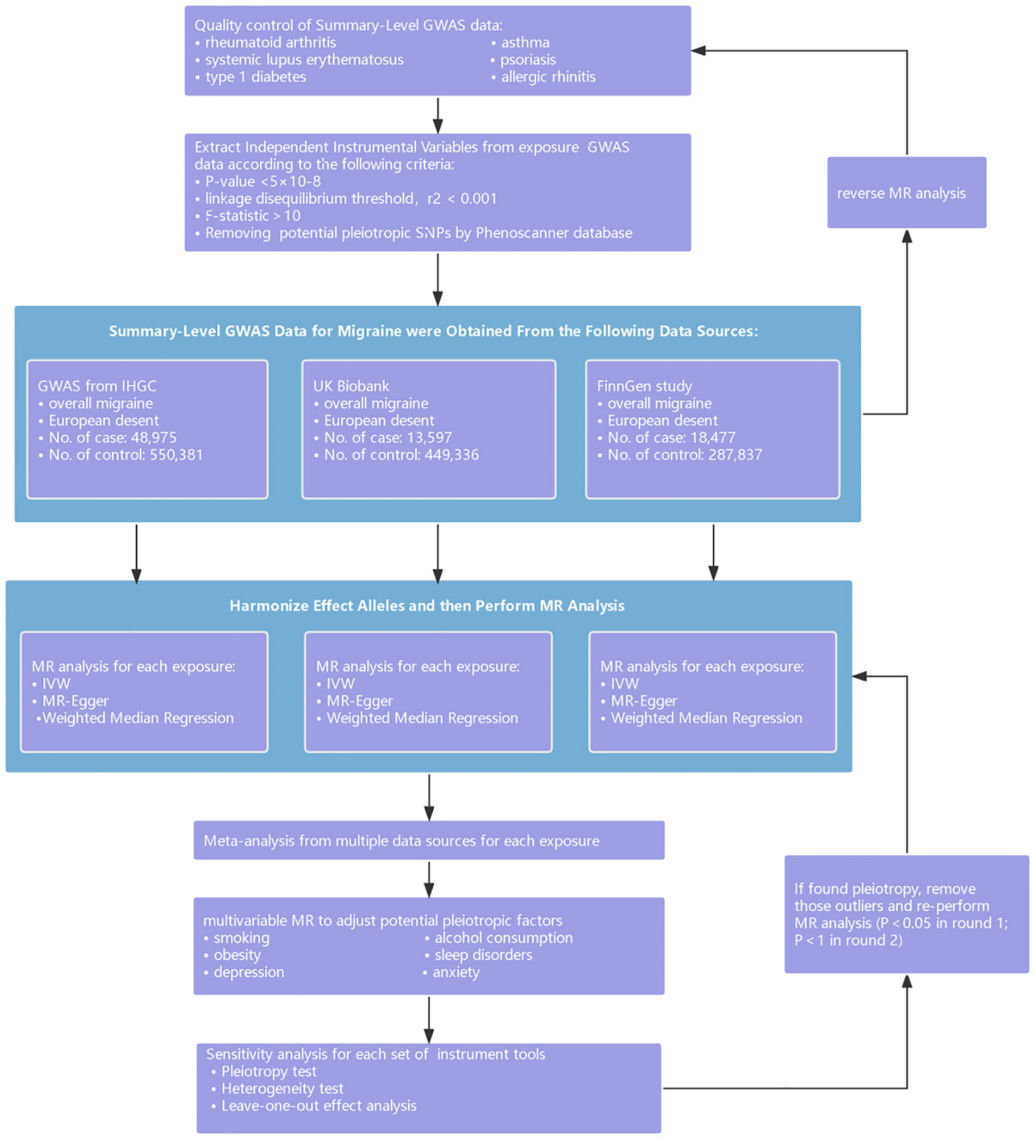
Schematic overview of the present study design. GWAS, genome-wide association study; MR, Mendelian randomization; IVW, inverse variance weighted; IHGC, International Headache Genetics Consortium.

First, we performed a two-sample MR analysis to identify causal relationships between immune diseases and migraine in the forward direction. Genetic IVs were employed as proxies for exposures to test the association with migraine and its major subtypes from 3 separate data sources, and then estimated the effect of exposures on outcomes. Second, reverse MR analysis was performed to determine whether migraine and its subtypes were risk factors for the immune disorders included in the current study. Third, we conducted multivariable MR (MVMR) of exposure to estimate whether immune disorders were independently associated with migraine by adjusting for potential pleiotropic factors, including smoking, alcohol consumption, obesity, sleep disorders, depression, and anxiety. In addition, we conducted a genetic correlation to identify shared genetic variants for these two associations. The summary-level data were obtained from publicly available GWAS studies or public databases. Such utilized data were already in the public domain, so no additional ethical approvals were required. The current study followed the Strengthening the Reporting of Observational Studies in Epidemiology using Mendelian Randomization (STROBE-MR) guidelines (Additional file 3, template available here: https://www.strobe-mr.org). A schematic overview of the present study design is presented in **Figure 1**.

### Data sources for migraine

Summary statistics for migraine were obtained from three separate data sources: GWAS meta-analyses from the International Headache Genetics Consortium (IHGC) (14), the UK Biobank, and the FinnGen consortium. Detailed information about data sources is shown in **Additional File 2**: **Supplementary Table 1**.

The IHGC is a population-level GWAS meta-analysis of 24 migraine cohorts from 5 study collections with a total sample of 102,084 cases and 771,257 controls. Because of privacy protection for participants, we had no access to the 23andMe cohort. Present GWAS summary statistics contain 599,356 European ancestry individuals (48,975 cases and 550,381 controls) after excluding the 23andMe cohort’s sample. Migraine cases were identified through clinical phenotyping or based on self-reported information. Genetic IVs for MO and MA were identified from earlier GWAS meta-analyses (23), respectively, including 14,7970 individuals (8,348 cases and 139,622 controls, European descent) and 151,215 individuals (6,332 cases and 144,883 controls, European descent).

The UK Biobank Consortium is a prospective, large-scale biomedical database that collected health and genetic information on over 500,000 general UK populations between 2006 and 2010 (24). SNPs associated with migraine were identified from summary statistics of the UK Biobank on a broad migraine definition (13,597 cases and 449,336 controls). All analyses were restricted to European ancestry.

The migraine GWAS of the FinnGen was based on the FinnGen data R9 with a total of 306,314 participants (18,477 cases and 287,837 controls) of British ancestry. Genetic IVs for MA and MO were also extracted from the FinnGen (MA: 7,917 cases and 287,837 controls; MO: 6,730 cases and 287,837 controls).

### Data sources for immunological diseases

The estimate from a two-sample MR analysis is less biased, and any bias is in the direction of the null when risk factor and outcome data are taken from non-overlapping datasets. However, using gene clusters with partially overlapping participants resulted in different extents and directions of bias (25). To eliminate potential bias associated with sample overlap, we chose GWAS studies of six immunological diseases after excluding the UK Biobank and FinnGen cohorts. The genetic backgrounds of the study populations were of European ancestry to avoid bias associated with race-related confounders.

All included GWAS studies of immunological diseases are publicly available. We selected the largest study with replication when there were multiple available GWAS studies for a single trait. We extracted SNPs associated with each autoimmune disease in each independent GWAS study. Notably, the current research has no overlap between GWAS populations used for exposure and outcome analyses. Detailed data sources can be found in **Additional File 2**: **Supplementary Table 1**.

### Genetic instruments selection

We performed quality control procedures on all summary datasets: (1) removing SNPs for non-binary alleles; (2) deleted SNPs in the major histocompatibility complex region (chr6: 25.5–33.5 Mb); (3) excluded SNPs with minor alleles frequency (MAF) < 0.01; (4) Ambiguous SNPs with incongruent alleles and palindromic SNPs with an ambiguous strand were excluded.

Suitable IVs need to fulfill the three central assumptions of MR analysis. First, Genetic variants must be strongly associated with exposure. We selected independent SNPs related to exposure at genome-wide significance (P-value < 5×10^-8^) as potential IVs. We increased the P-value < 5×10^-6^ for AR due to no available SNPs. A linkage disequilibrium (LD) threshold of r^2^ < 0.001 in a window size of 10 Mb was used to exclude the interference of LD. By calculating the F-statistic, the existence of weak instrumental variable bias for the selected SNPs can be measured. The formula for calculating the F-statistic is R^2^ (N-2)/(1-R^2^). In this equation, N is the sample size of GWAS, and R^2^ refers to the proportion of cumulative explained variance by the IVs. F > 10 indicates that no weak IV bias exists, thus further validating the association assumption. Secondly, genetic variant(s) must be associated with the outcome exclusively through the exposure. When IVs can affect outcomes through other pathways, genetic pleiotropy exists. The identification of confounders lacks specific evaluation criteria. In this study, six confounders, including smoking status, alcohol consumption, sleep disorders, obesity, stroke, myocardial infarction, depression, and anxiety, were considered as potential confounders. SNPs associated with outcome and these confounders were removed from IVs to exclude potential horizontal pleiotropy by searching and reviewing the Phenoscanner database. Moreover, a series of sensitivity analyses were conducted to validate the MR analysis assumptions and discussed in detail later. Finally, genetic variants must be independent of any measured and unmeasured confounders. The MR study was conducted using GWAS data for analysis, so the assumption is problematic to test directly. However, given that genetic variants precede the formation of acquired confounders, the genetic effects of genetic variants may be less affected by acquired confounders.

### Statistical analysis

All analyses were performed using R version 4.3.2 statistical software. We employed the Bonferroni correction to control for false discovery rates. Estimates with p-value < 0.05/6 (p < 0.008) were considered as strong evidence for causal effects, whereas correlations with p-value < 0.05 but > 0.008 indicated a suggestive causal effect.

### Genetic correlations

We first estimated the shared genetic architectures between immune diseases and migraine using the linkage disequilibrium score regression method (LDSC) before MR analysis. Software and procedures for conducting LDSC are publicly available (https://github.com/bulik/ldsc). The results of genetic correlation were corrected by applying the Bonferroni-corrected threshold of p-value < 0.05/6 (p < 0.008).

### Mendelian randomization

SNPs selected as IVs were harmonized with effective alleles in the outcome, and then causal analysis was performed. Three methods were employed to estimate causal effects: the inverse-variance weighted (IVW) method, weighted median regression, and the MR-Egger method. The IVW method is the current primary method for MR analysis owing to its higher test efficacy. The random-effects IVW was used if Cochran’s Q test showed heterogeneity between SNPs. Otherwise, we used a fixed-effects IVW. The remaining two MR methods were used as secondary methods to ascertain the robustness of the primary results. The weighted median regression also provides reliable estimates of causal effects when the valid IVs exceed more than 50% of the weights. In addition, we converted the beta values obtained to odds ratios and calculated confidence intervals to account for the results better. This ratio is an estimate of the causal effect of exposure on the outcome when the MR assumptions are fulfilled. Reverse MR analysis was performed using the same methods to estimate whether migraine is a risk factor for immune diseases. Considering the potential genetic correlations between immune diseases, as well as similar associations in epidemic studies, we performed MVMR to obtain independent estimates of effect sizes of exposure on outcomes by adjusting for genetically predicted immune diseases. Smoking, alcohol consumption, obesity, sleep disorders, depression, and anxiety were considered as potential pleiotropic factors in MVMR analyses.

### Sensitivity analysis

We performed a series of sensitivity analyses to assess the reliability and stability of the MR analyses. MR-Egger intercept test and MR‐pleiotropy residual sum and outlier (MR‐PRESSO) global tests were employed to detect horizontal pleiotropy. MR-Egger intercepts are based on the same regression model using inverse variance weighted (IVW) analysis, which can assess whether IVs produce pleiotropic effects on outcomes that differ on average from zero and provide consistent estimates of causal effects under weaker assumptions (26). The MR-Egger intercepts term moves away from zero, indicating the presence of directional pleiotropy in the selected genetic variants. Furthermore, we employed the MR-PRESSO framework to detect and correct horizontal pleiotropy by excluding outliers. MR-PRESSO test is best appropriate when less than 50% of the instruments show horizontal pleiotropy. If the global MR-PRESSO test P-value < 0.05 indicated significant horizontal pleiotropy, we removed the outliers defined by P-value < 0.05 and repeated the MR analysis using the remaining IVs. If pleiotropy were still present, outliers with P-value < 1 would be removed. We could only draw an uncertain conclusion for estimates that remain horizontal pleiotropy after two rounds of MR analysis. MR-egger and IVW in Cochran’s Q statistic were applied to detect the heterogeneity of different genetic variants, which could provide evidence of heterogeneity due to pleiotropy or other causes. The “leave-one-out” method was employed to assess the potential impact of outlying and pleiotropic SNPs on the overall estimate. It was estimated by removing individual SNPs and recalculating the causal effect estimates using the remaining SNPs to identify whether a single SNP drives the selected effect.

### Meta-analysis of the estimates

Each specific exposure was performed separately in each migraine outcome database of GWAS of the IHGC study, UK Biobank, and FinnGen study. Then, we conduct a meta-analysis to yield estimates of the pooled causal effect for each immune disease on migraine. We calculated I^2^ to statistics the heterogeneity between the estimates from multiple data sources and the p-values from Cochran’s Q-tests. When heterogeneity was less than 50%, a fixed-effects model was applied to summarize the instrumental variable estimates for each exposure. If not, we use random-effects models (27).

## Results

### Statistics for instrumental variables

The summary information about instruments identified for immune disorders and migraine and its subtypes are available in **Additional File 2: Supplementary Table 1**. Detailed information about the genetic variants and their associations in the respective outcome databases are given in **Additional File 1**: **S1**–**S4**. The F-statistics of all genetic instruments were all above the threshold of weak instruments of 10, indicating no weak instrumental variable bias.

### Genetic correlations

Using the LDSC, we detected no genetic correlation between migraine and immune disorders, including RA, SLE, T1D, AR, asthma, and psoriasis. The detailed results of genetic correlations are shown in **Additional File 2: Supplementary Table 3**.

### Causal effects from immunological diseases to migraine

**Figure 2:**
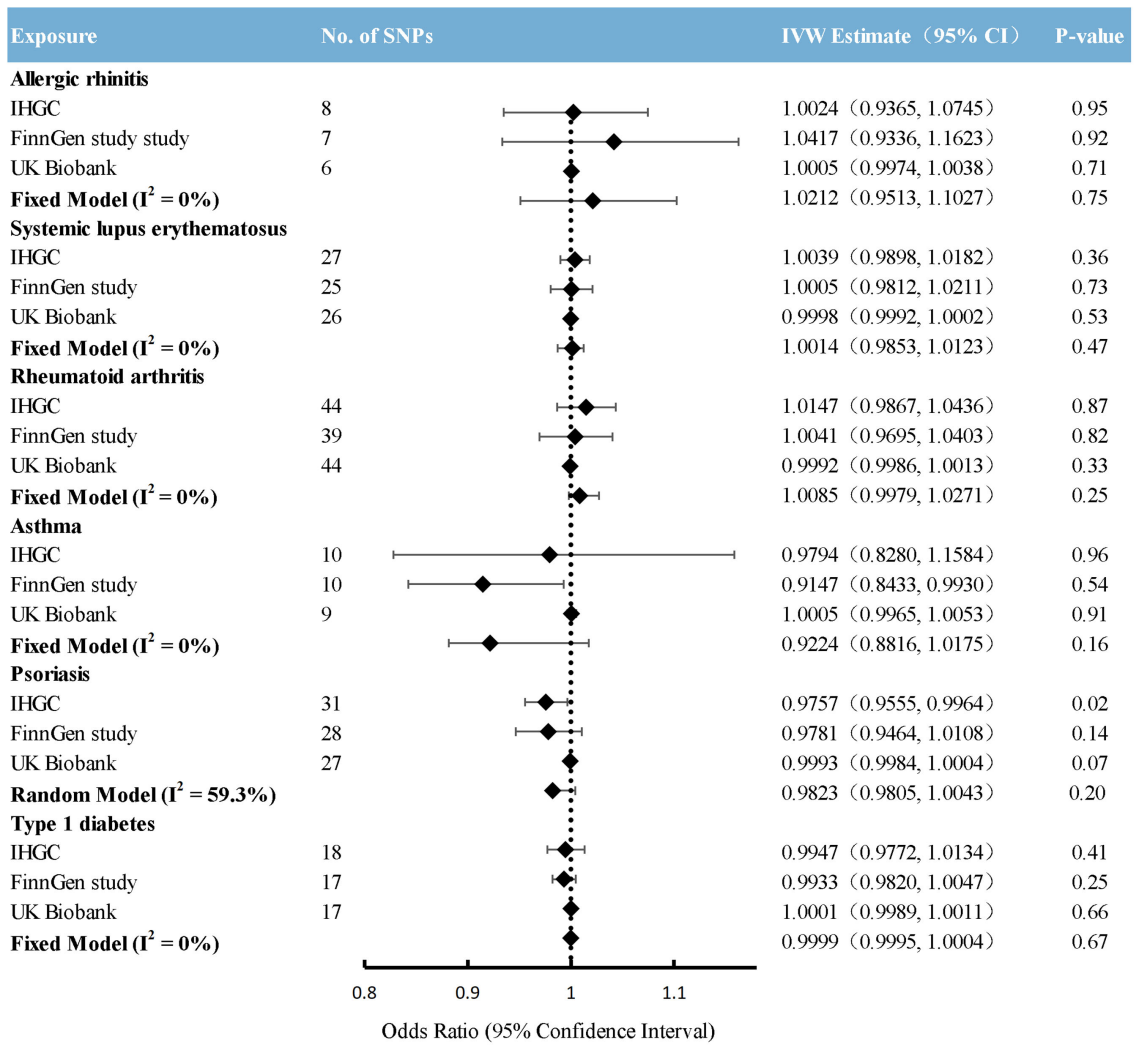
Causal association between immune diseases with migraine. Estimated ORs for the effect of immune diseases on migraine, obtained from an IVW analysis, per outcome database separately and combined over the 3 databases using fixed-effect meta-analyses when the heterogeneity was less than 50%: otherwise, using random-effects meta-analyses. CI, confidence interval; SNPs, single-nucleotide polymorphisms.

**Figure 3:**
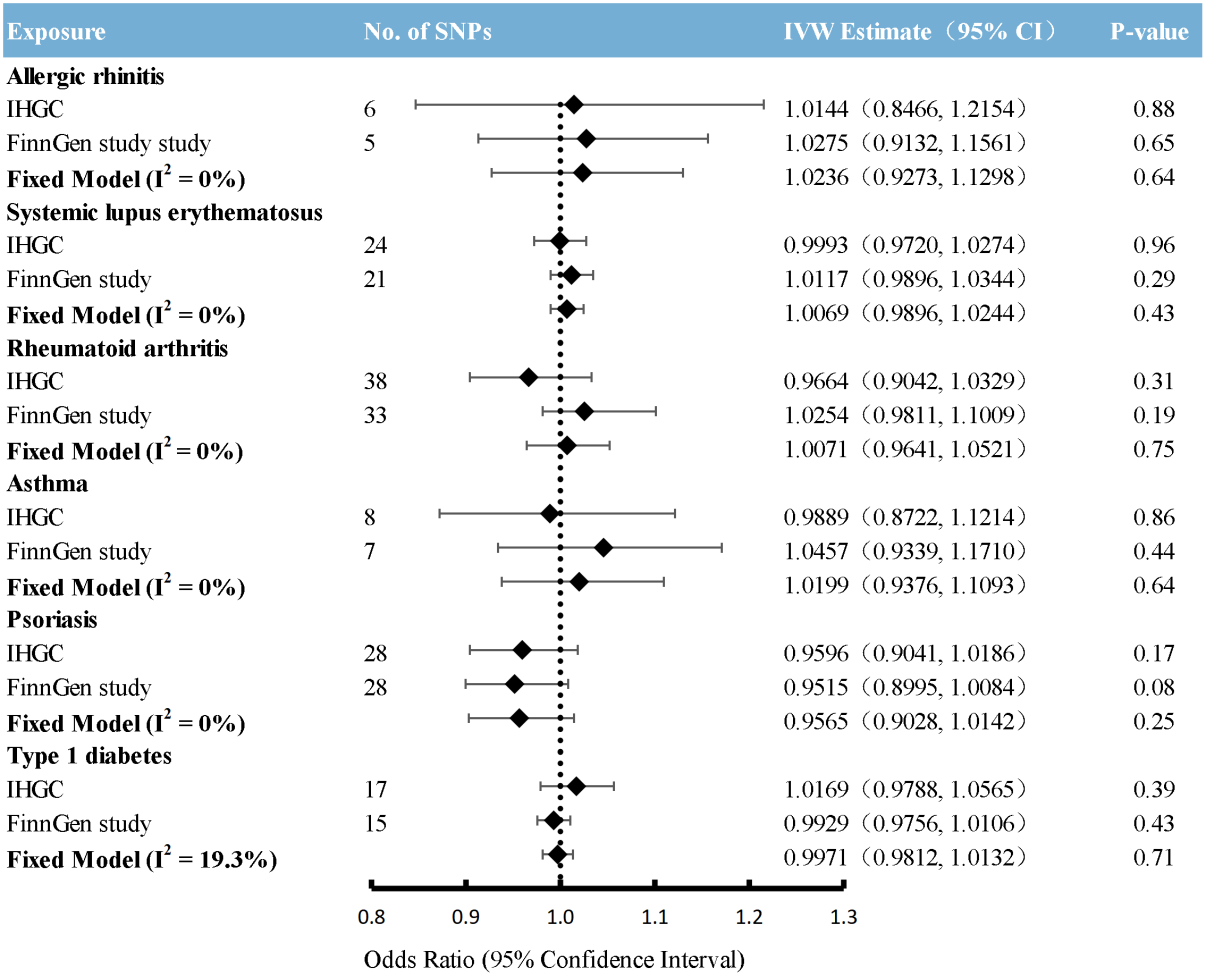
Causal association between immune diseases with migraine with aura. Estimated ORs for the effect of immune diseases on migraine with aura, obtained from an IVW analysis, per outcome database separately and combined over the 2 databases using fixed-effect meta-analyses when the heterogeneity was less than 50%: otherwise, using random-effects meta-analyses. CI, confidence interval; SNPs, single-nucleotide polymorphisms.

**Figure 4:**
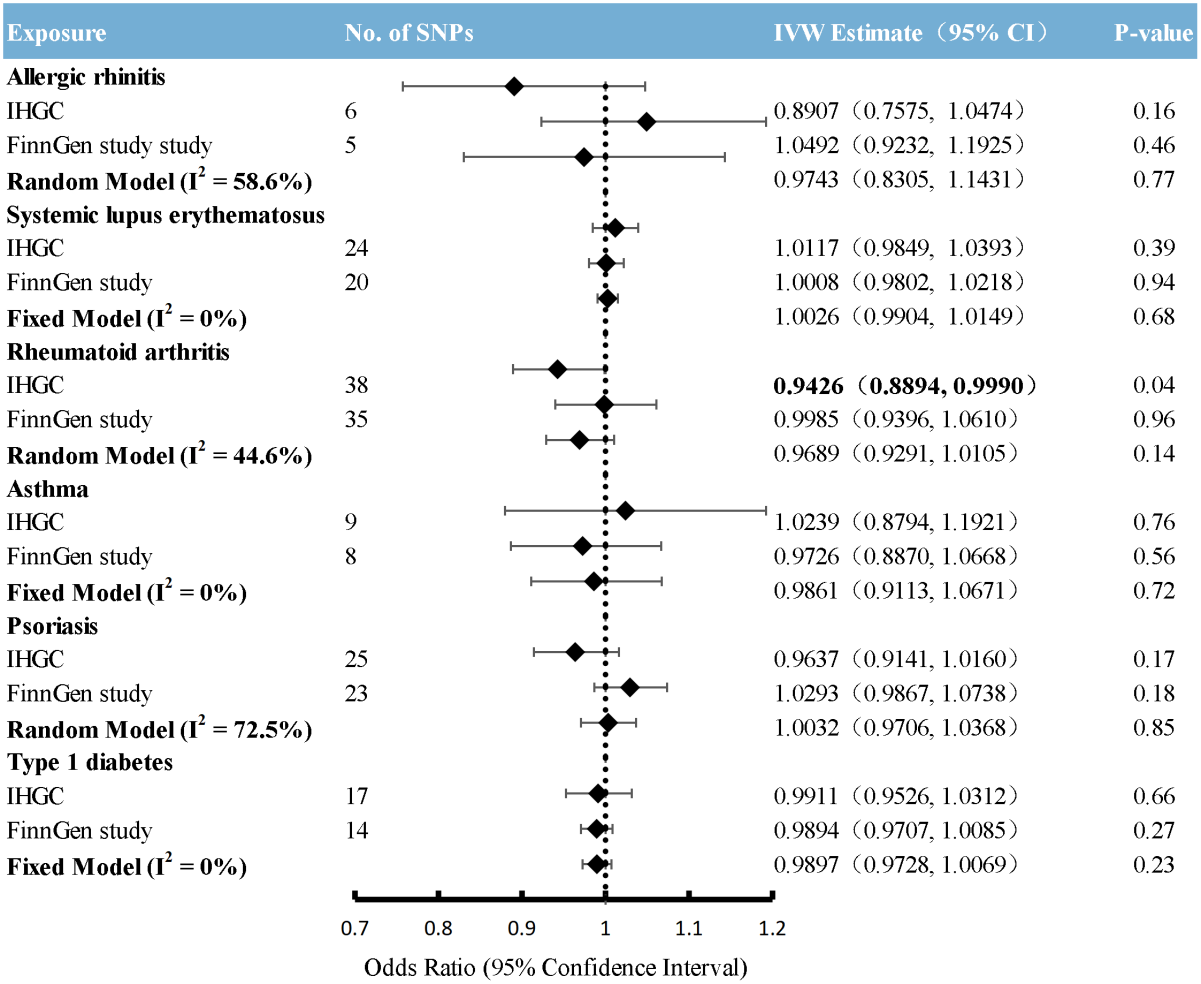
Causal association between immune diseases with migraine without aura. Estimated ORs for the effect of immune diseases on migraine without aura, obtained from an IVW analysis, per outcome database separately and combined over the 2 databases using fixed-effect meta-analyses when the heterogeneity was less than 50%: otherwise, using random-effects meta-analyses. CI, confidence interval; SNPs, single-nucleotide polymorphisms.

Overall, the primary MR analyses using IVW genetically determined immune diseases, including RA, SLE, T1D, AR, and asthma, were not associated with the risk of overall migraine when pooled in any of the three databases (**Figure 2**, **Additional File 2: Supplementary Table 4**). The findings of MR-Egger and WM were consistent. In general consistency with the findings for overall migraine, no associations were observed between the two main migraine subtypes, MA and MO, and genetically predicted immune disorders (**Figures 3**, **4**; **Additional File 2**: **Supplementary Tables 5**, **6**).

IVW analysis showed that only the result from the IHGC database indicated a suggestive association between psoriasis and overall migraine (P-value < 0.05). Yet, when pooling the results from three databases using a random-effects model, psoriasis and overall migraine risk became unrelated. Notably, after correcting multiple comparisons, psoriasis was not correlated with migraine. The estimates from weighted median regression and the MR-Egger method showed no evidence of a causal relationship between migraine and psoriasis.

Subsequently, we performed further MVMR analysis by removing potential pleiotropic factors to obtain an independent causal relationship between immune diseases and migraine (**Additional File 2: Supplementary Table 13**). The results of MVMR analyses indicated no genetic causality between immune disorders and migraine.

**Figure 5:**
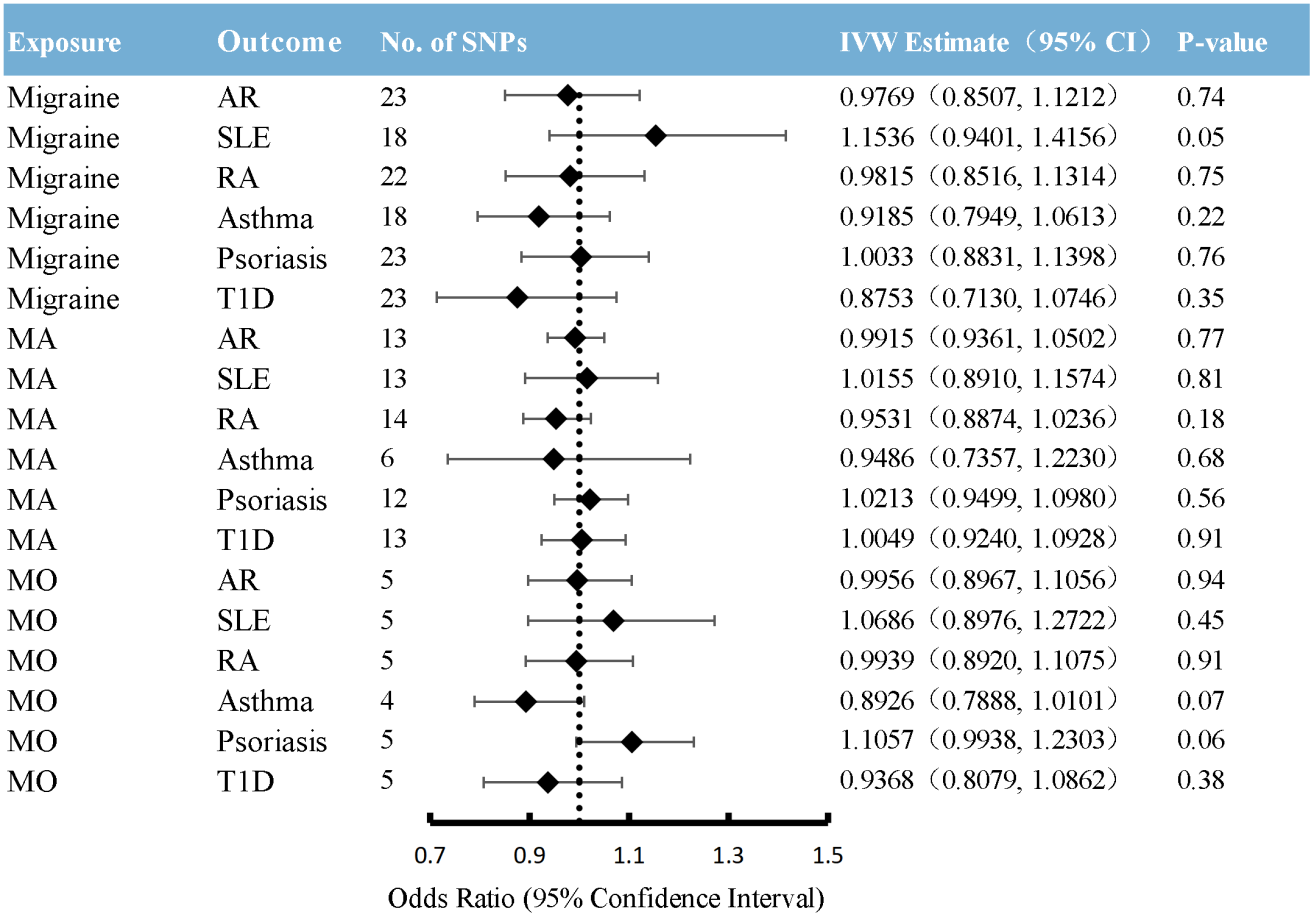
Causal association between migraine with immune diseases. Estimated ORs for the effect of migraine on immune diseases, obtained from an IVW analysis. CI: confidence interval; SNPs, single-nucleotide polymorphisms; AR, allergic rhinitis; SLE, systemic lupus erythematosus; RA, rheumatoid arthritis; T1D, type 1 diabetes; MA, migraine with aura; MO, migraine without aura.

In reverse MR, migraine was used as exposure, and six immune diseases were used as outcomes to explore the causal relationship. As shown in **Figure 5** and **Additional File 2: Supplementary Table 10**, no significant causal relationship was found between migraine and the risk of any immune diseases included in the present study. This finding was similar to weighted median regression and the MR-Egger method.

### Sensitivity analysis

MR-Egger intercept test suggested no significant deviation from zero for all analyses, implying no horizontal pleiotropy (**Additional File 2: Supplementary Tables 7**-**9**). In the MRPRESS analysis, significant horizontal pleiotropy (P-value < 0.05) was detected in five outcomes (**Additional File 2: Supplementary Table 12**), but no outlier SNP (all P-value > 0.05) was identified in any of the databases. We removed genetic variants (P-value < 1) detected in the MR-PRESSO analysis and repeated the MR analysis using the remaining IVs. The horizontal pleiotropy was controlled. The results of Cochran’s Q-test showed significant heterogeneity in some causal estimates. We did not perform the “leave-one-out” method because the current MR analysis did not identify an optimistic causal estimate.

## Discussion

The present study used the MR analysis to test whether immunological diseases are causally related to migraine risk and its two main subtypes. MR analyses for each outcome database were meta-analyzed to generate more accurate summary estimates. We also performed MVMR to obtain independent estimates of effect sizes of exposure on outcomes. Generally, MR analyses suggested that genetic predisposition of SLE, AR, RA, asthma, psoriasis, and T1D are unlikely to have a robust association with an increased risk of migraine (including overall migraine, MA, and MO), or vice versa.

The role of neuroinflammation and immune system derangement in migraine triggering has been widely discussed. Migraine begins with activation and sensitization of the trigeminovascular. Various neurotransmitters and neuropeptides, including calcitonin gene-related peptide (CGRP) and substance P (SP), are released (28, 29), causing an increased vascular permeability of the meninges, resulting in plasma protein leakage and leukocyte infiltration (30). Activation of immune cells by CGRP and SP causes mast cell degranulation (31), resulting in the release of multiple mediators such as histamine and serotonin. It selectively induces the release of pro-inflammatory cytokines and inflammatory compounds, including TNF-α, IL-1, and IL-6, ultimately contributing to neurogenic inflammation (32, 33). The role of pro-inflammatory cytokines in many autoimmune diseases is well known. Several pro-inflammatory cytokines, including TNF-α, IL-1β, IL-6, and IL-12p70 (34, 35) and the pro-inflammatory chemokine IL-8 (36), have been identified to increase significantly in peripheral levels in interictal migraine patients. Dysregulation of pro-inflammatory and anti-inflammatory factors in migraine may partially explain the pathophysiological association between migraine and immunological disorders.

The relationship between migraine and allergic diseases has been widely discussed. Several population-based studies with large samples, different age groups and countries have demonstrated the bidirectional epidemiological relationship between these diseases. Most studies reported a significant association between allergic disorders and migraine (37–41). Vincent and colleagues reported that immunotherapy of patients with allergic rhinitis showed reduced migraine prevalence, frequency, and disability (42). Only two retrospective questionnaire-based studies reported no association between asthma and migraine risk (43, 44). Despite the epidemiological association, the pathophysiological mechanisms underlying their relationship remain unclear. Neuroinflammation works in migraine attacks. Transient receptor potential vanilloid subfamily member 1 (TRPV1) expressed on trigeminal nerve injury receptors has been proven to play a key role in both migraine and asthma (45–47).TRPV1 releases neuropeptides, causing neurogenic inflammation, leading to vasodilation, and triggering migraine (48). The relationship between histamine and allergic reactions is widely known. Serum histamine levels were significantly higher in patients with migraine than in controls, and all patients with migraine had significantly higher histamine levels during headache than between non-headache periods (49). Histamine may affect hypothalamic function, mediating the pain response and regulation of headache attacks.

Generally, the current study showed no direct genetic causality between immunological diseases and migraine. The results were proven to stabilize through a series of sensitivity analyses. One possible explanation for the association between immune disorders and migraine in epidemiological studies may be the presence of potential confounders. First, drug treatments might partially affect migraine risk in patients with immunological disorders. Currently, disease-modifying antirheumatic drugs (DMARDs) may control immune-mediated disease activity effectively; however, long-term use may increase the risk of headache. A narrative review examining the safety of hydroxychloroquine indicated that headache was the most common extracutaneous adverse effect occurring within four weeks of initiating hydroxychloroquine (50). Up to 37% of patients receiving the immunosuppressant cyclosporine reported intermittent, mild, and diffuse headaches; few patients had migraine or a positive family history of migraine before taking cyclosporine. Reducing the cyclosporine dose relieved headaches in most patients (51). Secondly, comorbidities in patients with immune disorders may increase migraine risk. Obesity (52), insulin resistance (53), stressful life events (54), anxiety, and depression (52) are widely accepted as independent risk factors for migraine. These comorbidities are also widespread in many immunological disorders, which may contribute to an overestimation of the association between immunological disorders and migraine in observational studies. In addition, environmental triggers, such as weather and seasonal changes, foods, exercise, emotional stimulation, and chemicals, may trigger allergic diseases and migraines, which undoubtedly partially increase the association between migraine and allergic diseases. Finally, GWAS studies have attempted to explain the complex genetic structure of traits partly. However, immune disorders are inherently a remarkably heterogeneous disease group, with significant variations in pathophysiology and genetic susceptibility in immune disease patients from different ancestral backgrounds. The assumption that some alleles have not yet been sequenced may stand, which could lead to critical causative genes, remains undiscovered.

### Strengths and limitations

The present study has three main strengths, which enhance the validity and reliability of the results. Three data sources were meta-analyzed for gene-outcome correlations in this study, encompassing almost all available European population-based summary statistics on migraine except for the 23andMe cohort. Results from the three databases were generally consistent, enhancing the accuracy and reliability of the MR estimates. Secondly, using MVMR allowed us to explore independent causal estimates of the association between immune disorders and migraine. In addition, we used multiple sensitivity analyses to demonstrate the robustness of the results, which showed no pleiotropic effects.

The following limitations need to be considered. First, all the GWAS populations were of European ancestry. Though it reduced the bias induced by population stratification, to some extent, the findings may not be generalizable to other ethnic groups. Second, migraine cases were defined by ICD codes or self-reports, which may result in lower reliability for phenotype categorization. Third, considering the size of the immune disease GWAS, it is possible that some SNPs associated with these diseases have not yet been sequenced. Thus, the null results in the current study may be overturned when more significant alleles are identified. Finally, the recent null results may only represent the overall effect of genetically determined immune disorders on migraine, not the association of underlying specific biological or biomarker changes in immune disorders. Future studies should probably focus on identifying particular biomarkers of immune disease to provide more precise evidence for determining the relationship (55).

## Conclusion

To conclude, leveraging large-scale observational and genetic data of European ancestry, our findings do not support a causal association for SLE, AR, RA, asthma, psoriasis, and T1D with the risk of migraine and its subtypes, or vice versa. The current research may deepen our understanding of the biological mechanisms underlying the comorbidity of immune disorders and migraine. Studying potential central nervous system pathways and specific biomarker changes might assist in elucidating the fundamental link between immune disorders and migraine.

## Data availability statement

The original contributions presented in the study are included in the article/**Supplementary Material**. Further inquiries can be directed to the corresponding authors.

## Ethics statement

Ethical approval was not required for the studies involving humans because such utilized data were already in the public domain, so no additional ethical approvals were required. The studies were conducted in accordance with the local legislation and institutional requirements. Written informed consent for participation was not required from the participants or the participants’ legal guardians/next of kin in accordance with the national legislation and institutional requirements because such utilized data were already in the public domain, so no additional ethical approvals were required.

## Author contributions

GL: Conceptualization, Data curation, Formal analysis, Investigation, Methodology, Project administration, Software, Validation, Visualization, Writing – original draft, Writing – review & editing. SD: Conceptualization, Methodology, Supervision, Writing – review & editing. TZhe: Conceptualization, Methodology, Writing – review & editing. TZhu: Methodology, Supervision, Writing – review & editing. BQ: Methodology, Supervision, Writing – review & editing. LL: Funding acquisition, Resources, Supervision, Writing – review & editing. ZL: Funding acquisition, Project administration, Resources, Supervision, Writing – review & editing.

## References

[1] Ferrari MD Goadsby PJ Burstein R Kurth T Ayata C Charles A. Migraine. Nat Rev Dis Primers. (2022) 8:2. doi: 10.1038/s41572-021-00328-4 35027572

[2] Headache classification committee of the international headache society (IHS) the international classification of headache disorders, 3rd edition. Cephalalgia. (2018) 38:1–211. doi: 10.1177/0333102417738202 29368949

[3] Li G Duan S Zhu T Ren Z Xia H Wang Z. Efficacy and safety of intranasal agents for the acute treatment of migraine: a systematic review and network meta-analysis. J Headache Pain. (2023) 24:129. doi: 10.1186/s10194-023-01662-6 37723470 PMC10506288

[4] Feigin VL Vos T Alahdab F Amit A Barnighausen TW Beghi E. Burden of neurological disorders across the US from 1990-2017: A global burden of disease study. JAMA Neurol. (2021) 78:165–76. doi: 10.1001/jamaneurol.2020.4152 PMC7607495 33136137

[5] Global, regional, and national burden of neurological disorders during 1990-2015: a systematic analysis for the Global Burden of Disease Study 2015. Lancet Neurol. (2017) 16:877–97. doi: 10.1016/S1474-4422(17)30299-5 PMC5641502 28931491

[6] Grangeon L Lange KS Waliszewska-Prosol M Onan D Marschollek K Wiels W. Genetics of migraine: where are we now? J Headache Pain. (2023) 24:12. doi: 10.1186/s10194-023-01547-8 36800925 PMC9940421

[7] Charles AC Baca SM. Cortical spreading depression and migraine. Nat Rev Neurol. (2013) 9:637–44. doi: 10.1038/nrneurol.2013.192 24042483

[8] Hautakangas H Winsvold BS Ruotsalainen SE Bjornsdottir G Harder A Kogelman L. Genome-wide analysis of 102,084 migraine cases identifies 123 risk loci and subtype-specific risk alleles. Nat Genet. (2022) 54:152–60. doi: 10.1038/s41588-021-00990-0 PMC8837554 35115687

[9] Chen SP Fuh JL Chung MY Lin YC Liao YC Wang YF. Genome-wide association study identifies novel susceptibility loci for migraine in Han Chinese resided in Taiwan. Cephalalgia. (2018) 38:466–75. doi: 10.1177/0333102417695105 28952330

[10] Chang X Pellegrino R Garifallou J March M Snyder J Mentch F. Common variants at 5q33.1 predispose to migraine in African-American children. J Med Genet. (2018) 55:831–6. doi: 10.1136/jmedgenet-2018-105359 PMC6511513 30266756

[11] Choquet H Yin J Jacobson AS Horton BH Hoffmann TJ Jorgenson E. New and sex-specific migraine susceptibility loci identified from a multiethnic genome-wide meta-analysis. Commun Biol. (2021) 4:864. doi: 10.1038/s42003-021-02356-y 34294844 PMC8298472

[12] Bahrami S Hindley G Winsvold BS O'Connell KS Frei O Shadrin A. Dissecting the shared genetic basis of migraine and mental disorders using novel statistical tools. Brain. (2022) 145:142–53. doi: 10.1093/brain/awab267 PMC8967089 34273149

[13] Malik R Freilinger T Winsvold BS Anttila V Vander HJ Traylor M. Shared genetic basis for migraine and ischemic stroke: A genome-wide analysis of common variants. Neurology. (2015) 84:2132–45. doi: 10.1212/WNL.0000000000001606 PMC4451048 25934857

[14] Daghlas I Guo Y Chasman DI. Effect of genetic liability to migraine on coronary artery disease and atrial fibrillation: a Mendelian randomization study. Eur J Neurol. (2020) 27:550–6. doi: 10.1111/ene.14111 31661179

[15] Ngo ST Steyn FJ Mccombe PA. Gender differences in autoimmune disease. Front Neuroendocrinol. (2014) 35:347–69. doi: 10.1016/j.yfrne.2014.04.004 24793874

[16] Wang L Wang FS Gershwin ME. Human autoimmune diseases: a comprehensive update. J Intern Med. (2015) 278:369–95. doi: 10.1111/joim.12395 26212387

[17] Bigal ME Lipton RB. The epidemiology, burden, and comorbidities of migraine. Neurol Clin. (2009) 27:321–34. doi: 10.1016/j.ncl.2008.11.011 19289218

[18] Merikangas KR. Contributions of epidemiology to our understanding of migraine. Headache. (2013) 53:230–46. doi: 10.1111/head.12038 23432441

[19] Smith GD Hemani G. Mendelian randomization: genetic anchors for causal inference in epidemiological studies. Hum Mol Genet. (2014) 23:R89–98. doi: 10.1093/hmg/ddu328 PMC4170722 25064373

[20] Davies NM Holmes MV Smith GD. Reading Mendelian randomisation studies: a guide, glossary, and checklist for clinicians. BMJ. (2018) 362:k601. doi: 10.1136/bmj.k601 30002074 PMC6041728

[21] Lawlor DA Harbord RM Sterne JA Timpson N Smith GD. Mendelian randomization: using genes as instruments for making causal inferences in epidemiology. Stat Med. (2008) 27:1133–63. doi: 10.1002/sim.3034 17886233

[22] Sekula P Del Greco MF Pattaro C Kottgen A. Mendelian randomization as an approach to assess causality using observational data. J Am Soc Nephrol. (2016) 27:3253–65. doi: 10.1681/ASN.2016010098 PMC5084898 27486138

[23] Gormley P Anttila V Winsvold BS Palta P Esko T Pers TH. Meta-analysis of 375,000 individuals identifies 38 susceptibility loci for migraine. Nat Genet. (2016) 48:856–66. doi: 10.1038/ng.3598 PMC5331903 27322543

[24] Sudlow C Gallacher J Allen N Beral V Burton P Danesh J. UK biobank: an open access resource for identifying the causes of a wide range of complex diseases of middle and old age. PloS Med. (2015) 12:e1001779. doi: 10.1371/journal.pmed.1001779 25826379 PMC4380465

[25] Burgess S Davies NM Thompson SG. Bias due to participant overlap in two-sample Mendelian randomization. Genet Epidemiol. (2016) 40:597–608. doi: 10.1002/gepi.21998 27625185 PMC5082560

[26] Burgess S Thompson SG. Interpreting findings from Mendelian randomization using the MR-Egger method. Eur J Epidemiol. (2017) 32:377–89. doi: 10.1007/s10654-017-0255-x PMC5506233 28527048

[27] Luo J le Cessie S van Heemst D Noordam R. Diet-derived circulating antioxidants and risk of coronary heart disease: A Mendelian randomization study. J Am Coll Cardiol. (2021) 77:45–54. doi: 10.1016/j.jacc.2020.10.048 33413940

[28] Edvinsson L. Tracing neural connections to pain pathways with relevance to primary headaches. Cephalalgia. (2011) 31:737–47. doi: 10.1177/0333102411398152 21335366

[29] Ashina M. Migraine. N Engl J Med. (2020) 383:1866–76. doi: 10.1056/NEJMra1915327 33211930

[30] Spekker E Tanaka M Szabo A Vecsei L. Neurogenic inflammation: the participant in migraine and recent advancements in translational research. Biomedicines. (2021) 10. doi: 10.3390/biomedicines10010076 PMC8773152 35052756

[31] Lennerz JK Ruhle V Ceppa EP Neuhuber WL Bunnett NW Grady EF. Calcitonin receptor-like receptor (CLR), receptor activity-modifying protein 1 (RAMP1), and calcitonin gene-related peptide (CGRP) immunoreactivity in the rat trigeminovascular system: differences between peripheral and central CGRP receptor distribution. J Comp Neurol. (2008) 507:1277–99. doi: 10.1002/cne.21607 18186028

[32] Kulka M Sheen CH Tancowny BP Grammer LC Schleimer RP. Neuropeptides activate human mast cell degranulation and chemokine production. Immunology. (2008) 123:398–410. doi: 10.1111/j.1365-2567.2007.02705.x 17922833 PMC2433325

[33] Jansen-Olesen I Hougaard PS. PACAP and its receptors in cranial arteries and mast cells. J Headache Pain. (2018) 19:16. doi: 10.1186/s10194-017-0822-2 29460121 PMC5818390

[34] Uzar E Evliyaoglu O Yucel Y Ugur CM Acar A Guzel I. Serum cytokine and pro-brain natriuretic peptide (BNP) levels in patients with migraine. Eur Rev Med Pharmacol Sci. (2011) 15:1111–6. 22165670

[35] Biscetti L De Vanna G Cresta E Bellotti A Corbelli I Letizia CM. Immunological findings in patients with migraine and other primary headaches: a narrative review. Clin Exp Immunol. (2022) 207:11–26. doi: 10.1093/cei/uxab025 35020858 PMC8802184

[36] Duarte H Teixeira AL Rocha NP Domingues RB. Increased interictal serum levels of CXCL8/IL-8 and CCL3/MIP-1alpha in migraine. Neurol Sci. (2015) 36:203–8. doi: 10.1007/s10072-014-1931-1 25190547

[37] Sillanpaa M Aro H. Headache in teenagers: comorbidity and prognosis. Funct Neurol. (2000) 15 Suppl 3:116–21. 11200781

[38] Lateef TM Cui L Nelson KB Nakamura EF Merikangas KR. Physical comorbidity of migraine and other headaches in US adolescents. J Pediatr. (2012) 161:308–13.e1. doi: 10.1016/j.jpeds.2012.01.040 22381023 PMC4408276

[39] Ozge A Oksuz N Ayta S Uluduz D Yildirim V Toros F. Atopic disorders are more common in childhood migraine and correlated headache phenotype. Pediatr Int. (2014) 56:868–72. doi: 10.1111/ped.12381 24840677

[40] Turan MO Susuz CC Turan PA. Presence of headache and migraine in asthma patients. Turk Thorac J. (2017) 18:47–51. doi: 10.5152/TurkThoracJ. 29404159 PMC5783079

[41] Wei CC Lin CL Shen TC Chen AC. Children with allergic diseases have an increased subsequent risk of migraine upon reaching school age. J Investig Med. (2018) 66:1064–8. doi: 10.1136/jim-2018-000715 29903897

[42] Martin VT Taylor F Gebhardt B Tomaszewski M Ellison JS Martin GV. Allergy and immunotherapy: are they related to migraine headache? Headache. (2011) 51:8–20. doi: 10.1111/j.1526-4610.2010.01792.x 21054364

[43] Ronchetti R Villa MP Matricardi PM La Grutta S Barreto M Pagani J. Association of asthma with extra-respiratory symptoms in schoolchildren: two cross-sectional studies 6 years apart. Pediatr Allergy Immunol. (2002) 13:113–8. doi: 10.1034/j.1399-3038.2002.01036.x 12000483

[44] Becker C Brobert GP Almqvist PM Johansson S Jick SS Meier CR. The risk of newly diagnosed asthma in migraineurs with or without previous triptan prescriptions. Headache. (2008) 48:606–10. doi: 10.1111/j.1526-4610.2007.01030.x 18194300

[45] Kim JH. The emerging role of TRPV1 in airway inflammation. Allergy Asthma Immunol Res. (2018) 10:187–8. doi: 10.4168/aair.2018.10.3.187 PMC5911437 29676065

[46] Meents JE Neeb L Reuter U. TRPV1 in migraine pathophysiology. Trends Mol Med. (2010) 16:153–9. doi: 10.1016/j.molmed.2010.02.004 20347391

[47] Ferretti A Gatto M Velardi M Di Nardo G Foiadelli T Terrin G. Migraine, allergy, and histamine: is there a link? J Clin Med. (2023) 12. doi: 10.3390/jcm12103566 PMC10218803 37240671

[48] Benemei S De Cesaris F Fusi C Rossi E Lupi C Geppetti P. TRPA1 and other TRP channels in migraine. J Headache Pain. (2013) 14:71. doi: 10.1186/1129-2377-14-71 23941062 PMC3844362

[49] Gazerani P Pourpak Z Ahmadiani A Hemmati A Kazemnejad A. A correlation between migraine, histamine and immunoglobulin e. Scand J Immunol. (2003) 57:286–90. doi: 10.1046/j.1365-3083.2003.01216.x 12641658

[50] Gisondi P Piaserico S Bordin C Bellinato F Tozzi F Alaibac M. The safety profile of hydroxychloroquine: major cutaneous and extracutaneous adverse events. Clin Exp Rheumatol. (2021) 39:1099–107. doi: 10.55563/clinexprheumatol/styx9u 33635229

[51] Gijtenbeek JM van den Bent MJ Vecht CJ. Cyclosporine neurotoxicity: a review. J Neurol. (1999) 246:339–46. doi: 10.1007/s004150050360 10399863

[52] May A Schulte LH. Chronic migraine: risk factors, mechanisms and treatment. Nat Rev Neurol. (2016) 12:455–64. doi: 10.1038/nrneurol.2016.93 27389092

[53] Fava A Pirritano D Consoli D Plastino M Casalinuovo F Cristofaro S. Chronic migraine in women is associated with insulin resistance: a cross-sectional study. Eur J Neurol. (2014) 21:267–72. doi: 10.1111/ene.12289 24238370

[54] Scher AI Stewart WF Ricci JA Lipton RB. Factors associated with the onset and remission of chronic daily headache in a population-based study. Pain. (2003) 106:81–9. doi: 10.1016/S0304-3959(03)00293-8 14581114

[55] Yeung C Yeung SlAu Schooling CM. Association of autoimmune diseases with Alzheimer's disease: A mendelian randomization study. J Psychiatr Res. (2022) 155:550–8. doi: 10.1016/j.jpsychires.2022.09.052 36198219

