# Supplementary material for: Genetic causal relationship between immune diseases and migraine: a Mendelian randomization study
Source: Front Immunol. 2024 Apr 8;15:1376698. doi: 10.3389/fimmu.2024.1376698 (PMC11033421; doi:10.3389/fimmu.2024.1376698)
Supplement: Supplementary file 2 [file Table_2.docx]

## Supplementary Material

Supplementary table 1. Details data sources of the GWAS included in the Mendelian randomization.

Supplementary table 2. Potential pleiotropic SNPs removed by searching the Phenoscanner database.

Supplementary table 3. The results of genetic correlation between immune diseases and migraine

Supplementary table 4. MR estimates for the associations between immune diseases and migraine

# Supplementary table 5. MR estimates for the associations between immune diseases and migraine with aura.

Supplementary table 6. MR estimates for the associations between immune diseases and migraine without aura.

Supplementary table 7. Heterogeneity and Horizontal pleiotropy of MR analysis for immune diseases and migraine.

Supplementary table 8. Heterogeneity and Horizontal pleiotropy of MR analysis for immune diseases and migraine with aura.

Supplementary table 9. Heterogeneity and Horizontal pleiotropy of MR analysis for immune diseases and migraine without aura.

Supplementary table 10. MR estimates for the associations between migraine and immune diseases.

Supplementary table 11. Heterogeneity and Horizontal pleiotropy of MR analysis for migraine and immune diseases.

Supplementary table 12. Estimates from Mendelian randomization after excluding outlying genetic variants.

Supplementary table 13. Estimates from MVMR between immune diseases and migraine.

Supplementary table 1. Details of data sources of the GWAS included in the Mendelian randomization

| **Phenotype** | **Study type** | **No. of case** | **No. of control** | **Total No. of SNPs** | Population | Consortium/PubMed ID | Web Source |
| --- | --- | --- | --- | --- | --- | --- | --- |
| Rheumatoid arthritis | GWAS | 22,350 | 74,823 | 13,297,690 | European | 36333501 | GWAS-catalog: [GCST90132223](https://www.ebi.ac.uk/gwas/studies/GCST90132223) |
| SLE | Meta-analysis | 5,201 | 9,066 | 7,071,163 | European | 26502338 | IEU: ebi-a-GCST003156 |
| Type 1 diabetes | Meta-analysis | 9,266 | 15,574 | 12,783,129 | European | 32005708 | IEU: ebi-a-GCST010681 |
| Allergic rhinitis | GWAS | 13,936 | 42,701 | 14,515,094 | European | 33893285 | IEU: ebi-a-GCST90086040 |
| Asthma | Meta-analysis | 19,954 | 107,715 | 1,999,262 | European | 29273806 | IEU: ebi-a-GCST006862 |
| Psoriasis | GWAS | 15,967 | 28,194 | 8,536,277 | European | 34927100 | IEU: ebi-a-GCST90019016 |
| Overall Migraine | Meta-analysis | 48,975 | 550,381 | 11,380,305 | European | 35115687 | Dale Nyholt，d.nyholt@qut.edu.au |
|  | GWAS | 18,477 | 287,837 | 20,168,276 | European | FinnGen study | www.finngen.fi/en |
|  | GWAS | 13,597 | 449,336 | 9,851,867 | European | UK Biobank | IEU: ukb-b-16868 |
| Migraine with aura | Meta-analysis | 6,332 | 144,883 | 8,809,897 | European | 27322543 | if.iknisleh@cad-mmif |
|  | GWAS | 6,730 | 287,837 | 20,167,894 | European | FinnGen study | www.finngen.fi/en |
| Migraine without aura | Meta-analysis | 8,348 | 139,622 | 6,701,886 | European | 27322543 | if.iknisleh@cad-mmif |
|  | GWAS | 7,917 | 287,837 | 20,167,888 | European | FinnGen study | www.finngen.fi/en |
| Smoking initiation | GWAS | 311,629 | 321,173 | 11,802,365 | European | 30643251 | IEU: ieu-b-4877 |
| Alcohol consumption | GWAS | - | - | 11,887,865 | European | 30643251 | IEU: ieu-b-73 |
| Obesity | GWAS | 8,908 | 209,827 | 16,380,465 | European | FinnGen study | IEU: finn-b-E4_OBESITY |
| Sleep disorders | GWAS | 2,628 | 213,826 | 16,380,458 | European | FinnGen study | IEU: finn-b-F5_SLEEP |
| Depression | GWAS | 23,424 | 192,220 | 16,380,457 | European | FinnGen study | IEU: finn-b-F5_DEPRESSIO |
| Anxiety | GWAS | 12,513 | 198,110 | 16,380,449 | European | FinnGen study | IEU: finn-b-F5_ALLANXIOUS |

GWAS, genome-wide association study; SLE, systemic lupus erythematosus

Supplementary table 2. Potential pleiotropic SNPs removed by searching the Phenoscanner database.

| **Phenotypes** | **Removed SNPs** | **Reason for removal** |
| --- | --- | --- |
| SLE | rs389884 | Worrier or anxious feelings |
| SLE | rs597808 | Coronary artery disease, acute myocardial infarction, ever smoked |
| RA | rs12624433 | Anxiety, tension or depression |
| RA | rs2517951 | Bipolar disorder |
| Psoriasis | rs4889526 | Worrier or anxious feelings |
| T1D | rs10774624 | Coronary artery disease, myocardial infarction, Past tobacco smoking |
| T1D | rs10830227 | Seen doctor for nerves, anxiety, tension or depression |
| T1D | rs2111485 | Coronary artery disease |
| T1D | rs6679677 | Coronary artery disease |
| T1D | rs8056814 | Coronary artery disease |
| Asthma | rs7209400 | Coronary artery disease, self-reported heart attack or myocardial infarction |

SNPs, single-nucleotide polymorphisms; SLE, systemic lupus erythematosus; RA, rheumatoid arthritis; T1D, type 1 diabetes.

Supplementary table 3. The results of genetic correlation between immune diseases and migraine

| Phenotype | r_g_ | SE | Z score | P-value |
| --- | --- | --- | --- | --- |
| SLE | 0.0368 | 0.0363 | 1.0134 | 0.3108 |
| RA | 0.0124 | 0.0214 | 0.5779 | 0.5633 |
| Psoriasis | -0.0265 | 0.0165 | -1.605 | 0.1085 |
| T1D | -0.0201 | 0.0452 | -0.4436 | 0.6573 |
| Asthma | 0.0764 | 0.0438 | 1.7447 | 0.0810 |
| AR | 0.0864 | 0.1719 | 0.5025 | 0.6153 |

AR, allergic rhinitis; SLE, systemic lupus erythematosus; RA, rheumatoid arthritis; T1D, type 1 diabetes.

# Supplementary table 4. MR estimates for the associations between immune diseases and migraine

| **Exposure** | **Outcome** | **Data source** | **IVW** | | **Weighted median** | | **MR-Egger** | |
| --- | --- | --- | --- | --- | --- | --- | --- | --- |
|  |  |  | **Estimate （95% CI）** | **P-value** | **Estimate （95% CI）** | **P-value** | **Estimate （95% CI）** | **P-value** |
|  |  | IHGC | 1.0024（0.9365, 1.0745） | 0.95 | 1.0228（0.9363, 1.1178） | 0.60 | 1.0221（0.8337, 1.2530） | 0.34 |
|  |  | FinnGen study | 1.0417（0.9336, 1.1623） | 0.92 | 0.9823（0.8654, 1.1149） | 0.52 | 0.9460（0.7448, 1.2164） | 0.64 |
| AR | migraine | UK Biobank | 1.0005（0.9974, 1.0038） | 0.71 | 1.0007（0.9966, 1.0050） | 0.73 | 0.9866（0.9695, 1.0040） | 0.19 |
|  |  | Fixed Model | 1.0212（0.9513, 1.1027） | 0.75 | 1.0187（0.8965, 1.0849） | 0.74 | 0.9874（0.7904, 1.1846） | 0.15 |
|  |  | Random Model | 1.0212（0.9513, 1.1027） | 0.75 | 1.0187（0.8965, 1.0849） | 0.74 | 0.9874（0.7904, 1.1846） | 0.15 |
|  |  | IHGC | 1.0039（0.9898, 1.0182） | 0.36 | 1.0030（0.9850, 1.0214） | 0.14 | 1.0096（0.9771, 1.0432） | 0.21 |
| SLE | migraine | FinnGen study | 1.0005（0.9812, 1.0211） | 0.73 | 1.0024（0.9774, 1.0280） | 0.91 | 1.0095（0.9623, 1.0591） | 0.35 |
|  |  | UK Biobank | 0.9998（0.9992, 1.0002） | 0.53 | 0.9998（0.9992, 1.0004） | 0.65 | 0.9998（0.9990, 1.0007） | 0.73 |
|  |  | Fixed Model | 1.0014（0.9873, 1.0123） | 0.47 | 0.9998（0.9992, 1.0004） | 0.55 | 0.9998（0.9990, 1.0007） | 0.69 |
|  |  | Random Model | 1.0014（0.9873, 1.0023） | 0.47 | 1.0010（0.9957, 1.0064） | 0.70 | 1.0044（0.9928, 1.0162） | 0.45 |
|  |  | IHGC | 1.0147（0.9867, 1.0436） | 0.30 | 1.0157（0.9759, 1.0572） | 0.44 | 1.0874（0.9831, 1.2029） | 0.44 |
|  |  | FinnGen study | 1.0041（0.9695, 1.0403） | 0.82 | 1.0010（0.9423, 1.0633） | 0.97 | 1.0016（0.9243, 1.0856） | 0.45 |
|  |  | UK Biobank | 0.9988（0.9973, 1.0013） | 0.33 | 0.9994（0.9974, 1.0012） | 0.64 | 0.9987（0.9940, 1.0050） | 0.33 |
| RA | migraine | Fixed Model | 1.0085（0.9979, 1.0271） | 0.25 | **0.9967（0.9955, 0.9979）** | < 0.0001 | 1.0012（0.9979, 1.0045） | 0.73 |
|  |  | Random Model | 1.0085（0.9912, 1.0271） | 0.25 | 0.9810（0.9490, 1.0141） | 0.26 | 1.0012（0.9979, 1.0045） | 0.73 |
|  |  | IHGC | 0.9794（0.8280, 1.1584） | 0.96 | 1.0080（0.9465, 1.0736） | 0.75 | 1.3470（0.7867, 2.3065） | 0.70 |
| Asthma | migraine | FinnGen study | **0.9147（0.8426, 0.9930）** | **0.03** | 0.9322（0.8552, 1.0161） | 0.78 | 1.0634（0.8049, 1.4049） | 0.83 |
|  |  | UK Biobank | 1.0005（0.9965, 1.0053） | 0.91 | 0.9991（0.9973, 1.0018） | 0.60 | 0.9972（0.9823, 1.0124） | 0.68 |
|  |  | Fixed Model | 0.9217（0.8816, 1.0175） | 0.16 | 0.9993（0.9971, 1.0015） | 0.54 | 1.1973（0.9824, 1.2824） | 0.72 |
|  |  | Random Model | 0.9322（0.8992, 1.0173） | 0.16 | 1.0290（0.9626, 1.0999） | 0.40 | 1.1973（0.9824, 1.2824） | 0.72 |
|  |  | IHGC | **0.9757（0.9555, 0.9964）** | 0.02 | 0.9693（0.9390, 1.0006） | 0.44 | 0.9507（0.8995, 1.0048） | 0.71 |
|  |  | FinnGen study | 0.9781（0.9464, 1.0108） | 0.14 | 0.9797（0.9371, 1.0207） | 0.37 | 0.9374（0.8549, 1.0279） | 0.14 |
| Psoriasis | migraine | UK Biobank | 0.9993（0.9984, 1.0004） | 0.03 | 0.9997（0.9993, 1.0012） | 0.61 | 0.9995（0.9977, 1.0013） | 0.33 |
|  |  | Fixed Model | **0.9882（0.9790, 0.9998）** | 0.01 | 0.9997（0.9987, 1.0006） | 0.50 | 0.9995（0.9977, 1.0013） | 0.55 |
|  |  | Random Model | 0.9923（0.9805, 1.0043） | 0.20 | 0.9997（0.9987, 1.0006） | 0.50 | 0.9991（0.9943, 1.0040） | 0.73 |
|  |  | IHGC | 0.9947（0.9772, 1.0134） | 0.41 | 1.0052（0.9831, 1.0279） | 0.43 | 1.0133（0.9835, 1.0440） | 0.24 |
|  |  | FinnGen study | 0.9933（0.9820, 1.0047） | 0.25 | 0.9827（0.9756, 1.0070） | 0.28 | 0.9690（0.9389, 1.0001） | 0.06 |
| T1D | migraine | UK Biobank | 1.0001（0.9989, 1.0011） | 0.66 | 0.9995（0.9993, 1.0009） | 0.36 | 0.9998（0.9992, 1.0005） | 0.62 |
|  |  | Fixed Model | 0.9999（0.9995, 1.0004） | 0.67 | 0.9998（0.9993, 1.0003） | 0.43 | 0.9998（0.9991, 1.0004） | 0.53 |
|  |  | Random Model | 0.9999（0.9987, 1.0011） | 0.88 | 0.9998（0.9985, 1.0011） | 0.77 | 0.9981（0.9854, 1.0110） | 0.77 |

AR, allergic rhinitis; SLE, systemic lupus erythematosus; RA, rheumatoid arthritis; T1D, type 1 diabetes. Bold results indicate statistical significance.

# Supplementary table 5. MR estimates for the associations between immune diseases and migraine with aura

| **Exposure** | **Outcome** | **Data source** | **IVW** | | **Weighted median** | | **MR-Egger** | |
| --- | --- | --- | --- | --- | --- | --- | --- | --- |
|  |  |  | **Estimate （95% CI）** | **P-value** | **Estimate （95% CI）** | **P-value** | **Estimate （95% CI）** | **P-value** |
|  |  | IHGC | 1.0144（0.8466, 1.2154） | 0.88 | 1.0434（0.8208, 1.3263） | 0.73 | 1.0215（0.6291, 1.6586） | 0.93 |
| AR | MA | FinnGen study | 1.0275（0.9132, 1.1561） | 0.65 | 0.9934（0.8465, 1.1658） | 0.94 | 0.9243（0.7081, 1.2064） | 0.58 |
|  |  | Fixed Model | 1.0236（0.9273, 1.1298） | 0.64 | 1.0085（0.8828, 1.1521） | 0.90 | 0.9460（0.7490, 1.1948） | 0.64 |
|  |  | Random Model | 1.0236（0.9273, 1.1298） | 0.64 | 1.0085（0.8828, 1.1521） | 0.90 | 0.9460（0.7490, 1.1948） | 0.64 |
|  |  | IHGC | 0.9993（0.9720, 1.0274） | 0.96 | 0.9978（0.9579, 1.0394） | 0.92 | 1.0223（0.9590, 1.0899） | 0.50 |
| SLE | MA | FinnGen study | 1.0117（0.9896, 1.0344） | 0.29 | 1.0088（0.9797, 1.0389） | 0.55 | 1.0092（0.9615, 1.0594） | 0.71 |
|  |  | Fixed Model | 1.0069（0.9896, 1.0244） | 0.43 | 1.0050（0.9814, 1.0293） | 0.67 | 1.0140（0.9755, 1.0539） | 0.48 |
|  |  | Random Model | 1.0069（0.9896, 1.0244） | 0.43 | 1.0050（0.9814, 1.0293） | 0.67 | 1.0140（0.9755, 1.0539） | 0.48 |
|  |  | IHGC | 0.9664（0.9042, 1.0329） | 0.31 | 0.9354（0.8517, 1.0273） | 0.16 | 0.8259（0.6927, 0.9848） | 0.04 |
| RA | MA | FinnGen study | 1.0254（0.9811, 1.1009） | 0.32 | 1.0321（0.9438, 1.1287） | 0.48 | 1.0263（0.8985, 1.1722） | 0.70 |
|  |  | Fixed Model | 1.0071（0.9641, 1.0521） | 0.75 | 1.0250（0.9639, 1.0899） | 0.32 | 0.9484（0.8529, 1.0545） | 0.33 |
|  |  | Random Model | 1.0040（0.9352, 1.0779） | 0.91 | 1.0250（0.9639, 1.0899） | 0.49 | 0.9280（0.7505, 1.1475） | 0.49 |
|  |  | IHGC | 0.9889（0.8722, 1.1214） | 0.86 | 0.9824（0.8517, 1.1331） | 0.81 | 1.2478（0.8163, 1.9075） | 0.32 |
| Asthma | MA | FinnGen study | 1.0457（0.9339, 1.1710） | 0.44 | 1.0187（0.9089, 1.1418） | 0.75 | 0.9798（0.6524, 1.4714） | 0.92 |
|  |  | Fixed Model | 1.0199（0.9376, 1.1093） | 0.64 | 1.0044（0.9188, 1.0980） | 0.92 | 1.1000（0.8201, 1.4754） | 0.52 |
|  |  | Random Model | 1.0199（0.9376, 1.1093） | 0.64 | 1.0044（0.9188, 1.0980） | 0.92 | 1.1000（0.8201, 1.4754） | 0.52 |
|  |  | IHGC | 0.9596（0.9041, 1.0186） | 0.17 | 0.9876（0.9051, 1.0776） | 0.20 | 0.9096（0.7731, 1.0703） | 0.09 |
| Psoriasis | MA | FinnGen study | 0.9515（0.8995, 1.0084） | 0.08 | 0.9512（0.8885, 1.0184） | 0.01 | 0.9037（0.7734, 1.0558） | 0.14 |
|  |  | Fixed Model | 0.9565（0.9028, 1.0142） | 0.25 | 0.9794（0.9020, 1.0413） | 0.12 | 0.9053（0.7719, 1.0684） | 0.09 |
|  |  | Random Model | 0.9540（0.9028, 1.0142） | 0.25 | 0.9794（0.9020, 1.0413） | 0.12 | 0.9053（0.7719, 1.0684） | 0.09 |
|  |  | IHGC | 1.0169（0.9788, 1.0565） | 0.39 | 1.0134（0.9553, 1.0751） | 0.66 | 1.0292（0.9571, 1.1068） | 0.44 |
| T1D | MA | FinnGen study | 0.9929（0.9756, 1.0106） | 0.43 | 0.9938（0.9712, 1.0170） | 0.60 | 0.9848（0.9591, 1.0112） | 0.27 |
|  |  | Fixed Model | 0.9971（0.9812, 1.0132） | 0.71 | 0.9964（0.9752, 1.0180） | 0.73 | 0.9899（0.9656, 1.0148） | 0.42 |
|  |  | Random Model | 0.9986（0.9789, 1.0187） | 0.88 | 0.9964（0.9752, 1.0180） | 0.73 | 0.9932（0.9599.1.0277） | 0.69 |

SLE, systemic lupus erythematosus; RA, rheumatoid arthritis; T1D, type 1 diabetes; MA, migraine with aura. Bold results indicate statistical significance.

# Supplementary table 6. MR estimates for the associations between immune diseases and migraine without aura

| **Exposure** | **Outcome** | **Data source** | **IVW** | | **Weighted median** | | **MR-Egger** | |
| --- | --- | --- | --- | --- | --- | --- | --- | --- |
|  |  |  | **Estimate （95% CI）** | **P-value** | **Estimate （95% CI）** | **P-value** | **Estimate （95% CI）** | **P-value** |
|  |  | IHGC | 0.8907（0.7575, 1.0474） | 0.16 | 0.8510（0.6879, 1.0527） | 0.14 | 1.1255（0.7192, 1.7615） | 0.62 |
| AR | MO | FinnGen study | 1.0492（0.9232, 1.1925） | 0.46 | 1.1303（0.9501, 1.3448） | 0.17 | 1.1253（0.9327, 1.6626） | 0.18 |
|  |  | fixed Model | 0.9852（0.8911, 1.0893） | 0.77 | 1.0090（0.8820, 1.1543） | 0.89 | 1.1254（0.8827, 1.4347） | 0.34 |
|  |  | Random Model | 0.9743（0.8305, 1.1431） | 0.74 | 0.9876（0.7480, 1.3038） | 0.92 | 1.1254（0.8827, 1.4347） | 0.34 |
|  |  | IHGC | 1.0117（0.9849, 1.0393） | 0.39 | 1.0106（0.9727, 1.0500） | 0.59 | 1.0417（0.9732, 1.1149） | 0.25 |
| SLE | MO | FinnGen study | 1.0008（0.9802, 1.0218） | 0.94 | 1.0076（0.9788, 1.0374） | 0.61 | 1.0298（0.9846, 1.0771） | 0.21 |
|  |  | fixed Model | 1.0026（0.9904, 1.0149） | 0.68 | 1.0087（0.9856, 1.0323） | 0.73 | 1.0334（0.9954, 1.0728） | 0.08 |
|  |  | Random Model | 1.0026（0.9904, 1.0149） | 0.68 | 1.0087（0.9856, 1.0323） | 0.73 | 1.0334（0.9954, 1.0728） | 0.08 |
|  |  | IHGC | **0.9426（0.8894, 0.9990）** | 0.04 | 1.0032（0.9200, 1.0941） | 0.94 | 0.9581（0.8155, 1.1256） | 0.60 |
| RA | MO | FinnGen study | 0.9985（0.9396, 1.0610） | 0.96 | 1.0474（0.9601, 1.1427） | 0.30 | 1.0856（0.9461, 1.2457） | 0.25 |
|  |  | fixed Model | 0.9689（0.9291, 1.0105） | 0.14 | 1.0250（0.9639, 1.0899） | 0.43 | 1.0299（0.9276, 1.1435） | 0.58 |
|  |  | Random Model | 0.9695（0.9163.1.0257） | 0.28 | 1.0250（0.9639, 1.0899） | 0.43 | 1.0274（0.9098, 1.1602） | 0.66 |
|  |  | IHGC | 1.0239（0.8794, 1.1921） | 0.76 | 1.0343（0.9124, 1.1726） | 0.60 | 0.9816（0.5768, 1.6703） | 0.95 |
| Asthma | MO | FinnGen study | 0.9726（0.8870, 1.0668） | 0.56 | 0.9976（0.8868, 1.1223） | 0.97 | 1.1909（0.8695, 1.6313） | 0.29 |
|  |  | fixed Model | 0.9861（0.9113, 1.0671） | 0.72 | 1.0146（0.9311, 1.1056） | 0.74 | 1.1327（0.8640, 1.4849） | 0.37 |
|  |  | Random Model | 0.9861（0.9113, 1.0671） | 0.72 | 1.0146（0.9311, 1.1056） | 0.74 | 1.1327（0.8640, 1.4849） | 0.37 |
|  |  | IHGC | 0.9637（0.9141, 1.0160） | 0.17 | 0.9908（0.9261, 1.0600） | 0.79 | 1.0941（0.9527, 1.2566） | 0.21 |
| Psoriasis | MO | FinnGen study | 1.0293（0.9867, 1.0738） | 0.18 | 1.0169（0.9605, 1.0767） | 0.56 | 0.9333（0.8379, 1.0395） | 0.22 |
|  |  | fixed Model | 1.0032（0.9706, 1.0368） | 0.85 | 1.0059（0.9630, 1.0508） | 0.79 | 0.9910（0.9102, 1.0790） | 0.83 |
|  |  | Random Model | 0.9979（0.9357, 1.0643） | 0.95 | 1.0059（0.9630, 1.0508） | 0.79 | 1.0043（0.8598, 1.1730） | 0.95 |
|  |  | IHGC | 0.9911（0.9526, 1.0312） | 0.66 | 0.9919（0.9395, 1.0472） | 0.77 | 1.0156（0.9415, 1.0955） | 0.69 |
| T1D | MO | FinnGen study | 0.9894（0.9707, 1.0085） | 0.27 | 0.9883（0.9637, 1.0135） | 0.36 | 0.9791（0.9516, 1.0074） | 0.16 |
|  |  | fixed Model | 0.9897（0.9728, 1.0069） | 0.23 | 0.9889（0.9666, 1.0118） | 0.34 | 0.9836（0.9577, 1.0101） | 0.22 |
|  |  | Random Model | 0.9897（0.9728, 1.0069） | 0.23 | 0.9889（0.9666, 1.0118） | 0.34 | 0.9836（0.9577, 1.0101） | 0.22 |

SLE, systemic lupus erythematosus; RA, rheumatoid arthritis; T1D, type 1 diabetes; MO, migraine without aura

**Supplementary table 7. Heterogeneity and Horizontal pleiotropy of MR analysis for immune diseases and migraine**

| **Exposure** | **Outcome** | **Data source** | **Horizontal pleiotropy** | | **Heterogeneity test** | |
| --- | --- | --- | --- | --- | --- | --- |
|  |  |  | **MR_PRESSO Global P-value** | **Egger_intercept P-value** | **IVW_Q_P-val** | **Egger_Q_P-val** |
| AR | migraine | IHGC | 0.911 | 0.85 | 0.82 | 0.89 |
|  |  | FinnGen study | 0.144 | 0.57 | 0.12 | 0.09 |
|  |  | UK Biobank | 0.474 | 0.16 | 0.45 | 0.67 |
| SLE | migraine | IHGC | 0.090 | 0.76 | 0.08 | 0.07 |
|  |  | FinnGen study | 0.121 | 0.39 | 0.12 | 0.11 |
|  |  | UK Biobank | 0.664 | 0.21 | 0.65 | 0.61 |
| RA | migraine | IHGC | 0.331 | 0.44 | 0.34 | 0.33 |
|  |  | FinnGen study | 0.090 | 0.47 | 0.08 | 0.08 |
|  |  | UK Biobank | 0.063 | 0.15 | 0.07 | 0.09 |
| Asthma | migraine | IHGC | 0.352 | 0.34 | **0.02** | **0.01** |
|  |  | FinnGen study | 0.275 | 0.97 | **0.003** | **0.001** |
|  |  | UK Biobank | 0.362 | 0.64 | **2.92E-10** | **2.05E-10** |
| Psoriasis | migraine | IHGC | 0.374 | 0.66 | 0.36 | 0.33 |
|  |  | FinnGen study | 0.239 | 0.31 | 0.23 | 0.23 |
|  |  | UK Biobank | 0.214 | 0.93 | 0.21 | 0.18 |
| T1D | migraine | IHGC | 0.513 | 0.39 | 0.46 | 0.45 |
|  |  | FinnGen study | 0.474 | 0.11 | 0.42 | 0.51 |
|  |  | UK Biobank | 0.141 | 0.72 | 0.11 | 0.09 |

SLE, systemic lupus erythematosus; RA, rheumatoid arthritis; T1D, type 1 diabetes; AR, allergic rhinitis; IHGC, the International Headache Genetics Consortium

**Supplementary table 8. Heterogeneity and Horizontal pleiotropy of MR analysis for immune diseases and migraine with aura**

| **Exposure** | **Outcome** | **Data source** | **Horizontal pleiotropy** | | | **Heterogeneity test** | | |
| --- | --- | --- | --- | --- | --- | --- | --- | --- |
|  |  |  | **MR_PRESSO Global P-value** | | **Egger_intercept P-value** | **IVW_Q_P-val** | | **Egger_Q_P-val** |
| AR | MA | IHGC | 0.67 | 0.98 | | 0.66 | 0.54 | |
|  |  | FinnGen study | 0.55 | 0.41 | | 0.53 | 0.51 | |
| SLE | MA | IHGC | 0.34 | 0.44 | | 0.32 | 0.33 | |
|  |  | FinnGen study | 0.08 | 0.91 | | 0.08 | 0.06 | |
| RA | MA | IHGC | 0.32 | 0.06 | | 0.32 | 0.41 | |
|  |  | FinnGen study | 0.04 | 0.84 | | **0.003** | **0.003** | |
| Asthma | MA | IHGC | 0.05 | 0.28 | | **0.04** | 0.05 | |
|  |  | FinnGen study | 0.08 | 0.75 | | **0.006** | **0.004** | |
| Psoriasis | MA | IHGC | 0.38 | 0.09 | | 0.41 | 0.48 | |
|  |  | FinnGen study | 0.05 | 0.55 | | **0.04** | **0.04** | |
| T1D | MA | IHGC | 0.98 | 0.70 | | 0.97 | 0.97 | |
|  |  | FinnGen study | 0.35 | 0.42 | | 0.32 | 0.29 | |

SLE, systemic lupus erythematosus; RA, rheumatoid arthritis; T1D, type 1 diabetes; AR, allergic rhinitis; IHGC, the International Headache Genetics Consortium; MA, migraine with aura

**Supplementary table 9. Heterogeneity and Horizontal pleiotropy of MR analysis for immune diseases and migraine without aura**

| **Exposure** | **Outcome** | **Data source** | **Horizontal pleiotropy** | | **Heterogeneity test** | |
| --- | --- | --- | --- | --- | --- | --- |
|  |  |  | **MR_PRESSO Global P-value** | **Egger_intercept P-value** | **IVW_Q_P val** | **Egger_Q_P val** |
| AR | MO | IHGC | 0.68 | 0.31 | 0.64 | 0.68 |
|  |  | FinnGen study | 0.63 | 0.24 | 0.64 | 0.73 |
| SLE | MO | IHGC | 0.05 | 0.24 | **0.02** | **0.02** |
|  |  | FinnGen study | 0.65 | 0.17 | 0.62 | 0.67 |
| RA | MO | IHGC | 0.44 | 0.83 | 0.45 | 0.41 |
|  |  | FinnGen study | 0.18 | 0.19 | **0.01** | **0.02** |
| Asthma | MO | IHGC | 0.81 | 0.87 | **2.93E-05** | **1.60E-05** |
|  |  | FinnGen study | 0.27 | 0.21 | 0.25 | 0.3 |
| Psoriasis | MO | IHGC | 0.05 | 0.06 | **0.04** | 0.07 |
|  |  | FinnGen study | 0.11 | 0.06 | 0.12 | 0.19 |
| T1D | MO | IHGC | 0.12 | 0.46 | 0.11 | 0.10 |
|  |  | FinnGen study | 0.38 | 0.34 | 0.32 | 0.32 |

SLE, systemic lupus erythematosus; RA, rheumatoid arthritis; T1D, type 1 diabetes; AR, allergic rhinitis; IHGC, the International Headache Genetics Consortium; MO, migraine without aura

**Supplementary table 10. MR estimates for the associations between migraine and immune diseases**

| **Exposure** | **Outcome** | **No. of SNPs** | **IVW** |  | **Weighted median** |  | **MR-Egger** |  |
| --- | --- | --- | --- | --- | --- | --- | --- | --- |
|  |  |  | **Estimate （95% CI）** | **P-value** | **Estimate （95% CI）** | **P-value** | **Estimate （95% CI）** | **P-value** |
| Migraine | AR | 23 | 0.9769（0.8507, 1.1212） | 0.74 | 1.0224（0.8611, 1.2140） | 0.99 | 1.2480（0.8440, 1.8455） | 0.71 |
| Migraine | SLE | 18 | 1.1536（0.9401, 1.4156） | 0.05 | 1.1247（0.8579, 1.4743） | 0.30 | 1.0704（0.6261, 1.8197） | 0.97 |
| Migraine | RA | 22 | 0.9815（0.8516, 1.1314） | 0.75 | 1.0109（0.8614, 1.1863） | 0.78 | 0.9122（0.6084, 1.3677） | 0.65 |
| Migraine | Asthma | 18 | 0.9185（0.7949, 1.0613） | 0.22 | 0.8889（0.7760, 1.0183） | 0.09 | **0.6535（0.4443, 0.9631）** | **0.04** |
| Migraine | Psoriasis | 23 | 1.0033（0.8831, 1.1398） | 0.76 | 0.9676（0.8081, 1.1585） | 0.80 | 1.1111（0.7793, 1.5843） | 0.77 |
| Migraine | T1D | 23 | 0.8753（0.7130, 1.0746） | 0.35 | 0.8372（0.6772, 1.0350） | 0.29 | 0.5751（0.3304, 1.0011） | 0.28 |
| MA | AR | 13 | 0.9915（0.9361, 1.0502） | 0.77 | 1.0138（0.9358, 1.0983） | 0.73 | 0.9787（0.8736, 1.0964） | 0.71 |
| MA | SLE | 13 | 1.0155（0.8910, 1.1574） | 0.81 | 0.9633（0.8150, 1.1385） | 0.66 | 0.8826（0.6772, 1.1503） | 0.36 |
| MA | RA | 14 | 0.9531（0.8874, 1.0236） | 0.18 | 0.9680（0.8883, 1.0549） | 0.45 | 0.9502（0.8141, 1.1091） | 0.52 |
| MA | Asthma | 6 | 0.9486（0.7357, 1.2230） | 0.68 | 0.9093（0.7646, 1.0814） | 0.28 | 1.3661（0.3867, 4.8257） | 0.65 |
| MA | Psoriasis | 12 | 1.0213（0.9499, 1.0980） | 0.56 | 1.0425（0.9418, 1.1540） | 0.42 | 1.0211（0.8745, 1.1921） | 0.79 |
| MA | T1D | 13 | 1.0049（0.9240, 1.0928） | 0.91 | 1.0116（0.9013, 1.1354） | 0.84 | 1.0995（0.9261, 1.3054） | 0.29 |
| MO | AR | 5 | 0.9956（0.8967, 1.1056） | 0.94 | 1.0015（0.8844, 1.1340） | 0.98 | 1.1059（0.6874, 1.7793） | 0.69 |
| MO | SLE | 5 | 1.0686（0.8976, 1.2722） | 0.45 | 1.0130（0.8029, 1.2782） | 0.91 | 0.9205（0.3419, 2.4781） | 0.87 |
| MO | RA | 5 | 0.9939（0.8920, 1.1075） | 0.91 | 0.9619（0.8435, 1.0969） | 0.56 | 1.2974（0.7122, 2.3632） | 0.43 |
| MO | Asthma | 4 | 0.8926（0.7888, 1.0101） | 0.07 | 0.9019（0.7956, 1.0225） | 0.10 | 0.5089（0.1713, 1.5116） | 0.31 |
| MO | Psoriasis | 5 | 1.1057（0.9938, 1.2303） | 0.06 | 1.1489（0.9978, 1.3228） | 0.05 | 1.1827（0.7169, 1.9515） | 0.53 |
| MO | T1D | 5 | 0.9368（0.8079, 1.0862） | 0.38 | 0.8840（0.7340, 1.0647） | 0.19 | 0.6980（0.3011, 1.6178） | 0.43 |

SLE, systemic lupus erythematosus; RA, rheumatoid arthritis; T1D, type 1 diabetes; AR, allergic rhinitis; MA, migraine with aura; MO, migraine without aura. Bold results indicate statistical significance.

**Supplementary table 11. Heterogeneity and Horizontal pleiotropy of MR analysis for migraine and immune diseases**

| **Exposure** | **Outcome** | **Horizontal pleiotropy** | | **Heterogeneity test** | |
| --- | --- | --- | --- | --- | --- |
|  |  | **MR_PRESSO Global P-value** | **Egger_intercept P-value** | **IVW_Q_P_val** | **Egger_Q_P_val** |
| Migraine | AR | 0.78 | 0.60 | **0.04** | **0.04** |
| Migraine | SLE | 0.67 | 0.43 | 0.67 | 0.65 |
| Migraine | RA | 0.29 | 0.72 | 0.02 | **0.01** |
| Migraine | Asthma | 0.73 | 0.06 | **0.0002** | **0.0002** |
| Migraine | Psoriasis | 0.30 | 0.66 | 0.29 | 0.25 |
| Migraine | T1D | 0.05 | 0.07 | **0.04** | 0.08 |
| MA | AR | 0.38 | 0.79 | 0.39 | 0.34 |
| MA | SLE | 0.09 | 0.25 | 0.10 | 0.11 |
| MA | RA | 0.06 | 0.96 | 0.06 | 0.05 |
| MA | Asthma | 0.75 | 0.59 | **2.01131E-05** | **1.9521E-05** |
| MA | Psoriasis | 0.77 | 0.99 | 0.76 | 0.71 |
| MA | T1D | 0.28 | 0.25 | 0.28 | 0.30 |
| MO | AR | 0.97 | 0.67 | 0.98 | 0.97 |
| MO | SLE | 0.53 | 0.77 | 0.53 | 0.42 |
| MO | RA | 0.13 | 0.41 | 0.16 | 0.16 |
| MO | Asthma | 0.18 | 0.38 | 0.12 | 0.14 |
| MO | Psoriasis | 0.62 | 0.79 | 0.62 | 0.51 |
| MO | T1D | 0.28 | 0.51 | 0.24 | 0.21 |

SLE, systemic lupus erythematosus; RA, rheumatoid arthritis; T1D, type 1 diabetes; AR, allergic rhinitis; MA, migraine with aura; MO, migraine without aura. Bold results indicate the presence of heterogeneity.

**Supplementary table 12.** Estimates from Mendelian randomization after excluding outlying genetic variants

| **Exposure** | **Outcome** | **Data source** | **MR_PRESSO** | **Global P-value** | **No. of SNPs** | **Estimate （95% CI）** | **P-value** |
| --- | --- | --- | --- | --- | --- | --- | --- |
| SLE | Migraine | IHGC | initial result | **0.04** | 41 | 1.0051 (0.9941, 1.0163) | 0.36 |
|  |  |  | excluding outlying SNP | 0.09 | 39 | 1.0057 (0.9946, 1.0169) | 0.31 |
| SLE | MO | IHGC | initial result | **0.02** | 39 | 1.0035 (0.9740, 1.0039) | 0.82 |
|  |  |  | excluding outlying SNP | 0.17 | 37 | 1.0117 (0.9849, 1.0393) | 0.39 |
| RA | MA | FinnGen study | initial result | **0.004** | 62 | 1.0391 (0.9807, 1.1011) | 0.19 |
|  |  |  | excluding outlying SNP | 0.33 | 60 | 1.0254 (0.9811, 1.1009) | 0.32 |
| Asthma | MA | FinnGen study | initial result | **0.009** | 17 | 0.9814 (0.8621, 1.1173) | 0.77 |
|  |  |  | excluding outlying SNP | 0.05 | 16 | 1.0457 (0.9339, 1.1710) | 0.44 |
| Psoriasis | MO | IHGC | initial result | **0.04** | 52 | 0.9919 (0.8719, 1.1479) | 0.33 |
|  |  |  | excluding outlying SNP | 0.85 | 50 | 0.9637 (0.9141, 1.0160) | 0.17 |

SLE, systemic lupus erythematosus; RA, rheumatoid arthritis; T1D, type 1 diabetes; AR, allergic rhinitis; MA, migraine with aura; MO, migraine without aura. If the global MR-PRESSO test P value < 0.05 indicated significant horizontal pleiotropy, we removed the outliers with P < 0.05 and repeated the MR analysis using the remaining IVs. If pleiotropy were still present, outliers with P < 1 would be removed.

**Supplementary table 13.** Estimates from MVMR between immune diseases and migraine

| **Exposure** | **Outcome** | **Estimate （95% CI）** | **P-value** |
| --- | --- | --- | --- |
| Psoriasis | Migraine | 0.9840 (0.9571,1.0118) | 0.60 |
| Asthma | Migraine | 1.0039 (0.9493,1.0617) | 0.16 |
| T1D | Migraine | 0.9894 (0.9658,1.0135) | 0.57 |
| SLE | Migraine | 1.0066 (0.9903,1.0232) | 0.79 |
| AR | Migraine | 0.9775 (0.9179,1.0411) | 0.46 |
| RA | Migraine | 1.0198 (0.9574,1.0862) | 0.12 |

SLE, systemic lupus erythematosus; RA, rheumatoid arthritis; T1D, type 1 diabetes; AR, allergic rhinitis; MVMR, multivariable Mendelian randomization. We performed further MVMR analysis to obtain an independent causal relationship between immune diseases and migraine by adjusting for potential pleiotropic factors, including smoking, alcohol consumption, obesity, sleep disorders, depression, and anxiety.
